# Impact of the society for vascular surgery/society of thoracic surgeons classification on prognosis and therapeutic strategies in acute Stanford type A aortic dissection

**DOI:** 10.3389/fcvm.2026.1774053

**Published:** 2026-03-24

**Authors:** Zhendan Wu, Yue Yuan, Chen Chai, Ya Zhang, Lei Wang, Qi Sun, Wendan Wang, Xianbo Deng, Long Wu, Zehai Tang

**Affiliations:** 1 Department of Emergency Medicine, Union Hospital, Tongji Medical College, Huazhong University of Science and Technology, Wuhan, China; 2 Department of Emergency Medicine, Renmin Hospital of Wuhan University, Wuhan, China; 3 Department of Emergency Medicine, Zhongnan Hospital of Wuhan University, Wuhan, China; 4 Department of Emergency Intensive Care Unit, Sir Run Run Shaw Hospital, Hangzhou, China; 5 Department of Critical Care Medicine, Henan Provincial People’s Hospital, Zhengzhou, China

**Keywords:** acute Stanford type A aortic dissection, primary entry tear, society for vascular surgery, society of thoracic surgeons, SVS/STS classification

## Abstract

**Objective:**

The Stanford classification does not differentiate between antegrade and retrograde primary entry tears in acute Stanford type A aortic dissection (ATAAD). The 2020 Society for Vascular Surgery/Society of Thoracic Surgeons (SVS/STS) classification categorizes ATAAD into SVS/STS-A and SVS/STS-B0 based on the location of the primary entry tear; however, its clinical utility requires further validation. This study aims to evaluate the impact of the SVS/STS-A and SVS/STS-B0 on the prognosis and strategies for ATAAD.

**Methods:**

We retrospectively reviewed 931 consecutive ATAAD patients at Union Hospital (Wuhan, China) from June 2012 to August 2025. According to the SVS/STS classification criteria, patients were classified as the SVS/STS-A (*n* = 743) and SVS/STS-B0 (*n* = 188) dissection. Baseline demographics, aortic anatomical features, treatment strategies, and clinical outcomes were compared between the two groups.

**Results:**

The SVS/STS-A patients had more severe proximal aortic pathology, while the SVS/STS-B0 patients had more extensive distal involvement. The SVS/STS-A group underwent more aortic root replacement (36.8% vs. 7.7%, *P* < 0.001), aortic valve replacement (35.7% vs. 9.9%, *P* < 0.001), and coronary artery bypass grafting (35.1% vs. 9.2%, *P* < 0.001). In contrast, the SVS/STS-B0 group underwent more total arch replacement (78.2% vs. 65.1%, *P* = 0.012) and the stented elephant trunk procedure (78.9% vs. 59.1%, *P* < 0.001). In the overall cohort, the SVS/STS-A group demonstrated higher 24 h mortality (9.8% vs. 1.1%, *P* < 0.001), in-hospital mortality (24.2% vs. 14.4%, *P* = 0.004), and 3-year all-cause mortality (31.70% vs. 17.80%, *P* = 0.0005) compared to the SVS/STS-B0 group. In the open surgery subgroup, no significant prognostic difference existed between the two types. In the non-surgery subgroup, the SVS/STS-A group had significantly higher 24 h mortality (31.6% vs. 5.7%, *P* = 0.002) and in-hospital mortality (49.5% vs. 8.6%, *P* < 0.001).

**Conclusions:**

The SVS/STS classification provides effective preoperative risk stratification for ATAAD. The SVS/STS-A dissections carry a higher early mortality risk, necessitating urgent surgical intervention with aggressive management of the aortic root. In contrast, the SVS/STS-B0 dissections allow a wider surgical window, with strategies emphasizing arch replacement and distal remodeling.

## 1 Introduction

Acute aortic dissection (AAD) is characterized by the tearing of the aortic intima, which allows blood to enter the medial layer of the aortic wall, leading to the separation of its various layers (1). It is a life-threatening cardiovascular emergency. According to the latest data from the International Registry of Acute Aortic Dissection (IRAD), the mortality rate for medically managed Stanford type A aortic dissection remains as high as 0.5% per hour, with a cumulative mortality rate reaching 23.7% within 48 h (2). In contrast, the mortality rate can be reduced to 4.4% for patients undergoing surgical repair (2). Therefore, precise risk stratification and personalized therapeutic strategies are critical for improving patient prognosis.

The Stanford classification, which categorizes aortic dissection merely based on whether the ascending aorta is involved (3, 4), fails to encompass the crucial element of the primary intimal tear location. This system cannot distinguish between antegrade and retrograde entry tears, the two types that may differ fundamentally in their pathophysiological mechanisms, therapeutic urgency, and management strategies (5). The DeBakey classification further subdivides dissection into three types based on the location of the primary entry tear and the extent of the dissection: Type I features an entry tear in the ascending aorta with distal propagation beyond the aortic arch; Type II is confined to the ascending aorta; and Type III originates in the descending thoracic aorta, potentially limited to this segment (IIIa) or extending into the abdominal aorta (IIIb) (6). However, this classification also fails to adequately categorize retrograde dissections with an entry tear located in the aortic arch or distal to it (6, 7). These classification inadequacies have driven the development of new classification systems that primarily use the location of the primary entry tear as the key discriminator (8).

**Figure 1:**
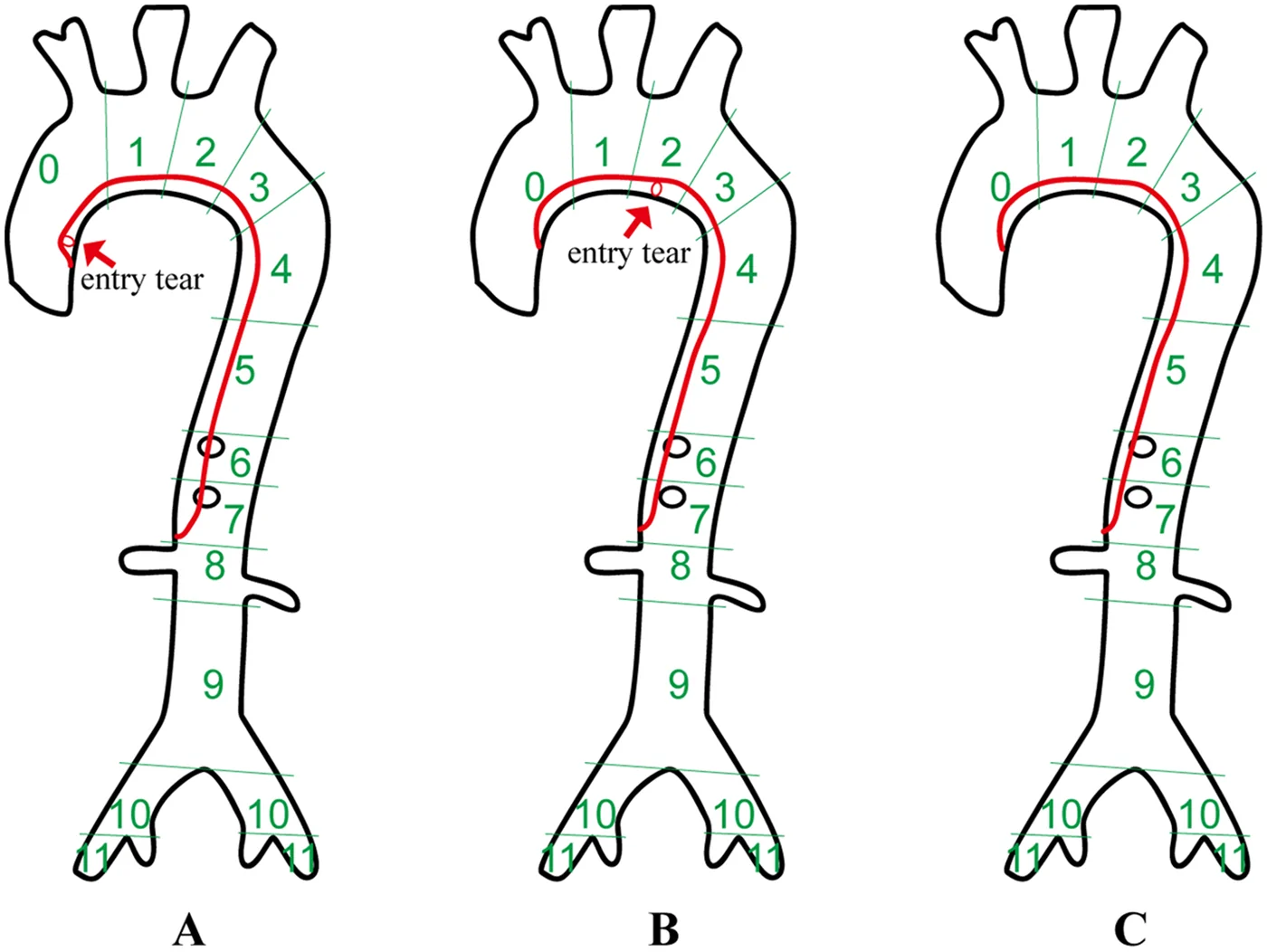
Schematic illustration of the SVS/STS aortic zoning system. **(A)** SVS/STS-A: entry tear in Zone 0. **(B)** SVS/STS-B0: entry tear distal to Zone 0 with retrograde extension into Zone 0. **(C)** SVS/STS-I: primary entry tear cannot be identified.

In 2020, the Society for Vascular Surgery (SVS) and the Society of Thoracic Surgeons (STS) jointly established a new classification system for aortic dissection based on the anatomic location of the intimal tear (8). This system divides the aorta into 11 distinct zones (Figure 1). A dissection with the primary entry tear located in Zone 0 (from the aortic root to distal to the innominate artery) is defined as SVS/STS-A. A dissection with the primary entry tear in Zone 1 or beyond is defined as SVS/STS-B, with the subtype B0 (the focus of this study) specifically denoting those in which the dissection process involves Zone 0 retrograde. Cases where the primary entry tear cannot be identified are classified as SVS/STS-I. This classification distinguishes between antegrade and retrograde ATAAD, yet its clinical value lacks validation in large-scale studies. Existing research predominantly focuses on surgical cohorts (9). This focus overlooks patients who die early in the disease course, potentially leading to an overestimation of surgical efficacy and an underestimation of the true risk associated with SVS/STS-A dissections.

Based on this background, the present study aims to: (1) systematically compare the differences between SVS/STS-A and SVS/STS-B0 dissections in the ATAAD cohort from Union Hospital, Wuhan, China; (2) elucidate the implications of the entry tear location for surgical management; and (3) evaluate the utility of this classification in prognostic evaluation. Ultimately, this research seeks to provide an evidence-based foundation for anatomy-driven precision medicine in the treatment of aortic dissection.

## 2 Materials and methods

### 2.1 Study design and population

**Figure 2:**
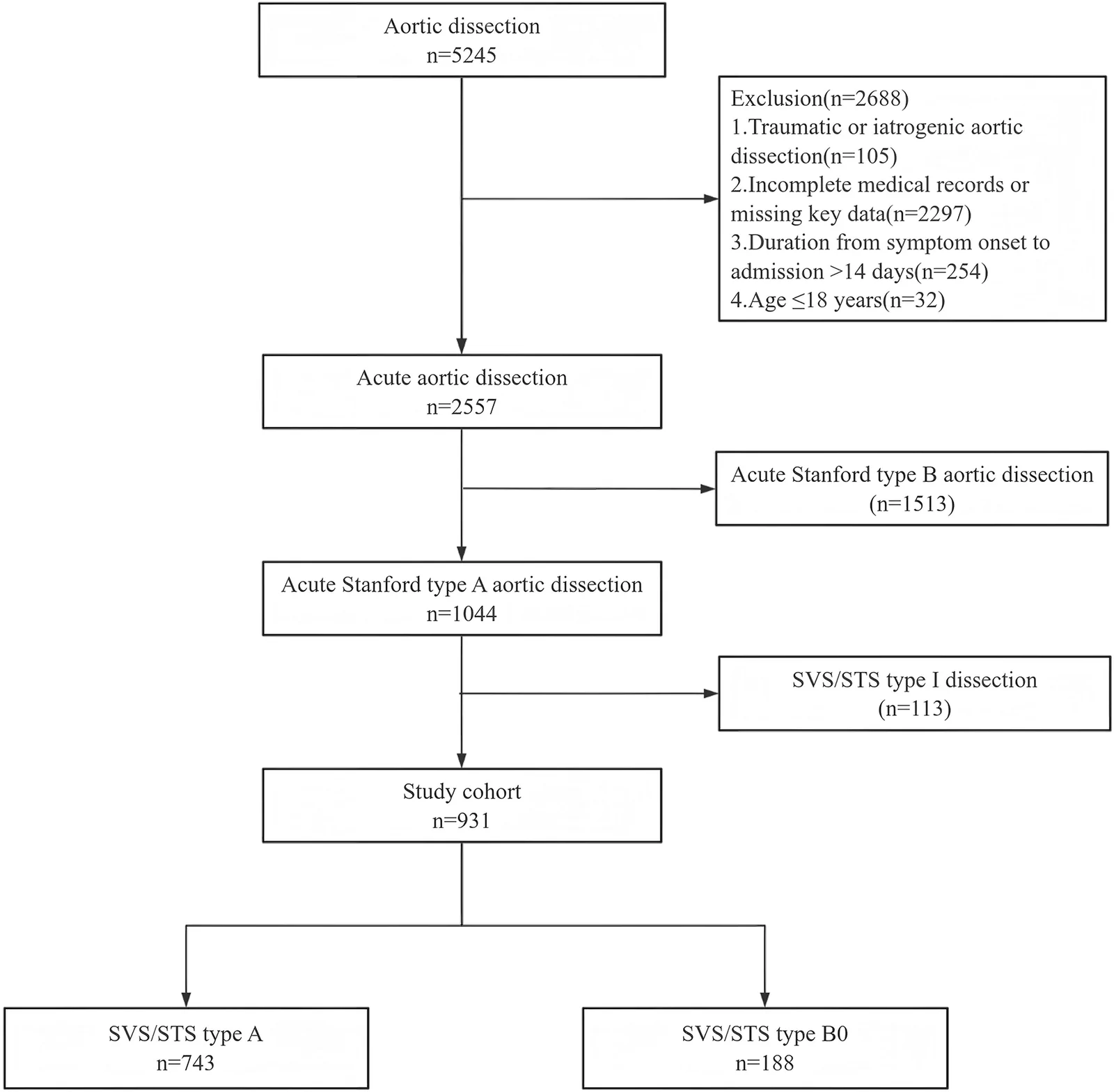
Inclusion and exclusion criteria for patient selection with SVS/STS-A and B0.

We conducted a retrospective cohort study at a single center. Consecutive patients (*n* = 5,245) admitted for aortic dissection (AD) to Union Hospital, Wuhan, China, between June 2012 and August 2025 were initially screened. After stepwise exclusions (Figure 2) (2,688 patients due to incomplete total aorta CTA data (*n* = 2,297), chronic dissection (*n* = 254), traumatic or iatrogenic aortic dissection (*n* = 105), or age ≤ 18 (*n* = 32), and 1,513 patients with acute Stanford type B AD), a total of 1,044 patients with ATAAD were included. Patients were stratified into SVS/STS-A (*n* = 743), SVS/STS-B0 (*n* = 188), and SVS/STS-I (*n* = 113) based on the SVS/STS classification system. SVS/STS types A/B/I were retrospectively assigned based on objective anatomical structures identified from preoperative computed tomography angiography (CTA) or magnetic resonance imaging (MRI) and operative records. During the study period, clinical decision-making and treatment strategies at our institution were based exclusively on the Stanford classification system, independent of the SVS/STS classification. This ensures that treatment allocation was unaffected by the SVS/STS categories, thereby mitigating potential treatment selection bias and enhancing methodological transparency. This study focused on patients with SVS/STS-A or SVS/STS-B0 dissections. SVS/STS-I patients were excluded from the primary comparative analysis but retained for separate outcome analysis (see Section 3.6). After excluding SVS/STS-I patients, the final study cohort comprised 931 patients (SVS/STS-A group: *n* = 743, 79.8%; SVS/STS-B0 group: *n* = 188, 20.2%).

Inclusion criteria: (1) Age > 18 years. (2) Symptom onset ≤ 14 days. (3) Diagnosis of Stanford type A aortic dissection confirmed by computed tomography angiography (CTA) or magnetic resonance imaging (MRI). (4) Availability of imaging data allowing clear identification of the primary entry tear location, meeting the Society for Vascular Surgery/Society of Thoracic Surgeons (SVS/STS) classification criteria. Exclusion criteria: (1) Traumatic or iatrogenic aortic dissection. (2) Incomplete clinical data or missing key variables. (3) Inability to determine the entry tear location. (4) Chronic aortic dissection (symptom onset > 14 days).

Patients were divided into three groups based on the final treatment strategy: the open surgery group, the endovascular treatment group, and the non-surgery group. The non-surgery group was further subdivided into four categories: (1) Death prior to planned intervention; (2) Presence of absolute surgical contraindications (e.g., massive cerebral infarction, intracerebral hemorrhage, active gastrointestinal bleeding, multiple organ failure, etc.); (3) Refusal of surgery by the patient or family; (4) Imaging findings indicating a predominantly thrombosed false lumen with stable clinical symptoms, managed with medical therapy alone.

### 2.2 Data collection

The data were sourced from the electronic medical record database: baseline characteristics [age, sex, height, weight and body mass index (BMI)], cardiovascular risk factors (diabetes mellitus, hypertension, smoking history and dyslipidemia), comorbidities (coronary artery disease, chronic obstructive pulmonary disease), vital signs at admission, and ancillary investigations (electrocardiographic findings, laboratory test results, imaging data). Treatment strategies and surgical details were recorded.

### 2.3 Outcome measures

The primary outcome was in-hospital all-cause mortality. Secondary outcomes included: early mortality (at 24 and 48 h), mid-term mortality (at 1 year and 3 years after admission), the incidence of in-hospital complications (aortic rupture, cardiac arrest, massive pericardial effusion,cardiac tamponade, cerebral infarction, spinal cord ischemia, acute renal failure), and days in hospital.

### 2.4 Statistical analysis

Survival curves were plotted using GraphPad Prism (v10.5), whereas other statistical analyses were performed using IBM SPSS Statistics (v31.0). For normally distributed continuous variables, data are presented as mean ± standard deviation (SD), and statistical comparison was performed using the *t*-test. For non-normally distributed continuous variables, data are presented as median (interquartile range, IQR), and statistical comparison was performed using the Mann–Whitney *U*-test. Categorical variables were expressed as number (percentage) and compared using the chi-square test or Fisher's exact test, as appropriate. All analyses used two-sided tests; *P*-values < 0.05 were considered statistically significant. Survival curves were generated using Kaplan–Meier analysis and compared using the log-rank test. The identification of independent risk factors was performed using multivariable Cox regression analysis.

### 2.5 CTA image interpretation

All preoperative computed tomography angiography (CTA) scans were independently reviewed by two specialists: an experienced radiologist and a senior emergency physician, both of whom were blinded to clinical outcomes and treatment assignments. Each reviewer assessed the following parameters as defined by the SVS/STS classification scheme: location of the primary entry tear, extent of dissection, involvement of branch vessels, and morphological features of the false lumen (including thrombosis and ulcer-like projections). Any disagreement between the two reviewers was resolved through direct discussion, with a third senior cardiac surgeon consulted if consensus could not be reached. This multidisciplinary approach aligns with contemporary aortic dissection imaging standards and enhances diagnostic reliability.

### 2.6 Ethical approval and quality control

This retrospective cohort study was approved by the Ethics Committee of Union Hospital, Tongji Medical College, Huazhong University of Science and Technology (Wuhan, China) (Approval No. 1005-01), which waived the need for individual informed consent.

## 3 Results

### 3.1 Baseline comparison

Baseline characteristics differed significantly between the SVS/STS-A and SVS/STS-B0 groups (Table 1). The difference in mean age between the two groups approached but did not reach statistical significance. The SVS/STS-B0 group had a higher proportion of male patients, a greater prevalence of smoking history, and a higher rate of hypertension compared to the SVS/STS-A group. Conversely, female sex and a history of coronary artery disease were more prevalent in the SVS/STS-A group. Mean body mass index (BMI) was also higher in the SVS/STS-B0 cohort. No significant intergroup differences were observed in diabetes mellitus, dyslipidemia, or chronic obstructive pulmonary disease.

**Table 1:** Baseline characteristics.

Variable	SVS/STS-A (*n* = 743)	SVS/STS-B0 (*n* = 188)	*P*-value
Age, years, mean ± SD	54.06 ± 12.89	52.03 ± 11.46	0.051
Female, *n* (%)	198 (26.6)	24 (12.8)	<0.001
BMI, kg/m², mean ± SD	25.12 ± 3.80	26.37 ± 3.75	<0.001
Smoking History, *n* (%)	240 (32.3)	83 (44.1)	0.002
Hypertension, *n* (%)	537 (72.3)	157 (83.5)	0.002
Diabetes Mellitus, *n* (%)	55 (7.4)	12 (6.4)	0.629
Dyslipidemia, *n* (%)	86 (11.6)	25 (13.3)	0.515
COPD, *n* (%)	43 (5.8)	9 (4.8)	0.594
Coronary Artery Disease, *n* (%)	138 (18.6)	20 (10.6)	0.010

COPD, chronic obstructive pulmonary disease; BMI, body mass index;.

### 3.2 Comparison of anatomical characteristics

The aortic anatomical features of the two groups displayed distinct patterns (Table 2). The SVS/STS-A group was characterized by a more pronounced burden of proximal aortic pathology, showing significantly higher rates of coronary ostia involvement (42.8% vs. 25.5%, *P* < 0.001), severe aortic regurgitation (31.6% vs. 10.6%, *P* < 0.001), and involvement of the innominate artery (70.7% vs. 54.8%, *P* < 0.001).

**Table 2:** Anatomical characteristics.

Anatomical Characteristic	SVS/STS-A (*n* = 743)	SVS/STS-B0 (*n* = 188)	*P*-value
Location of primary entry tear, *n* (%)			<0.001
Zone 0	743 (100.0)	0 (0)	
Zone 1	0 (0)	35 (18.6)	
Zone 2	0 (0)	48 (25.5)	
Zone 3	0 (0)	79 (42.1)	
Zone 4	0 (0)	19 (10.1)	
Zone 5	0 (0)	4 (2.1)	
Zone 6	0 (0)	3 (1.6)	
Branch involvement, *n* (%)			
Coronary artery	318 (42.8)	48 (25.5)	<0.001
Brachiocephalic trunk	525 (70.7)	103 (54.8)	<0.001
Left common carotid artery	362 (48.7)	76 (40.4)	0.042
Left subclavian artery	403 (54.2)	120 (63.8)	0.018
Celiac trunk	399 (53.7)	111 (59.0)	0.189
Superior mesenteric artery	329 (44.3)	89 (47.3)	0.451
Inferior mesenteric artery	268 (36.1)	92 (48.9)	0.001
Renal artery	399 (53.7)	133 (70.7)	<0.001
Common iliac artery	421 (56.7)	128 (68.1)	0.004
Internal iliac artery	204 (27.5)	66 (35.1)	0.039
External iliac artery	249 (33.5)	68 (36.2)	0.492
Thrombosed false lumen with ULP, *n* (%)	83 (11.2)	45 (23.9)	<0.001
Dissection extending below diaphragm, *n* (%)	517 (69.6)	164 (87.2)	<0.001
DeBakey type II, *n* (%)	75 (10.1)	0 (0.0)	<0.001
Severe aortic insufficiency, *n* (%)	235 (31.6)	20 (10.6)	<0.001

In contrast, although the SVS/STS-B0 group involved the ascending aorta through retrograde extension, it exhibited more extensive distal propagation of the dissection and greater branch vessel involvement. This included a higher frequency of extension below the diaphragm (87.2% vs. 69.6%, *P* < 0.001), as well as higher rates of renal artery (70.7% vs. 53.7%, *P* < 0.001), inferior mesenteric artery (48.9% vs. 36.1%, *P* = 0.001), and iliac artery involvement (68.1% vs. 56.7%, *P* = 0.004). Ulcer-like projection (*ULP*) with associated false lumen thrombosis was also observed more frequently among patients classified as SVS/STS-B0 (23.9% vs. 11.2%, *P* < 0.001).

### 3.3 Treatment modalities

#### 3.3.1 Treatment strategies

Treatment strategies differed significantly between the SVS/STS-A and SVS/STS-B0 groups (Table 3). The overall rate of surgical intervention (encompassing both open and endovascular therapy alone) was substantially higher in the SVS/STS-B0 (81.4% vs. 71.5%, *P* = 0.006).

**Table 3:** Initial treatment strategies.

Treatment strategy	SVS/STS-A (*n* = 743)	SVS/STS-B0 (*n* = 188)	*P-*value
Treatment Modality, *n* (%)			0.006
Surgical Intervention	531 (71.5)	153 (81.4)	
Non-Surgery	212 (28.5)	35 (18.6)	
Details of Surgery, *n* (%)	*n* = 531	*n* = 153	<0.001
Open surgery	530 (99.8)	142 (92.8)	
Endovascular therapy alone	1 (0.2)	11 (7.2)	
Details of Non-Surgery, *n* (%)	*n* = 212	*n* = 35	<0.001
Death before intervention	84 (39.6)	3 (8.6)	
Surgery declined	110 (51.9)	24 (68.6)	
Surgical contraindications	15 (7.1)	4 (11.4)	
Medical management alone	3 (1.4%)	4 (11.4)	

Among the 247 patients managed no-surgery, pre-intervention mortality was substantially higher in the SVS/STS-A [39.6% (84/212) vs. 8.6% (3/35) in the SVS/STS-B0 group, *P* < 0.001]. Within this non-surgery cohort, a small subset of patients—1.4% (3/212) in the SVS/STS-A and 11.4% (4/35) in the SVS/STS-B0 — presented with imaging evidence of a predominantly thrombosed false lumen alongside stable symptoms, and thus received medical therapy alone.

Open surgery was the primary treatment modality in 684 patients undergoing surgical intervention. In the SVS/STS-A group, 99.8% (530/531) underwent open surgery, while only 0.2% (1/531) received palliative abdominal aortic stent grafting. In the SVS/STS-B0 group, 81.4% (142/153) underwent open surgery, and 7.2% (11/153) received endovascular therapy alone (*P* < 0.001).

#### 3.3.2 Details of open surgical procedures

Among the 672 patients treated with open surgery, operative approaches differed significantly between the SVS/STS-A and SVS/STS-B0 group (Table 4). SVS/STS-A patients underwent aortic root replacement (36.8% vs. 7.7%, *P* < 0.001), aortic valve replacement (35.7% vs. 9.9%, *P* < 0.001), and coronary artery bypass grafting (35.1% vs. 9.2%, *P* < 0.001) more frequently. Conversely, the SVS/STS-B0 group showed higher rates of total aortic arch replacement (78.2% vs. 65.1%, *P* = 0.012) and stented elephant trunk procedures (78.9% vs. 59.1%, *P* < 0.001).

**Table 4:** Open surgical details.

Open Surgical Details	SVS/STS-A (*n* = 530)	SVS/STS-B0 (*n* = 142)	*P*-value
Aortic Root Management, *n* (%)			<0.001
Root Replacement	195 (36.8)	11 (7.7)	
Root Repair	93 (17.5)	22 (15.5)	
Aortic Valve Management, *n* (%)			<0.001
Valve Replacement	189 (35.7)	14 (9.9)	
Valve Repair/Plasty	75 (14.2)	15 (10.6)	
Aortic Arch Management, *n* (%)			0.012
Total Arch Replacement	345 (65.1)	111 (78.2)	
Hemiarch Replacement	50 (9.4)	8 (5.6)	
Elephant Trunk, *n* (%)	313 (59.1)	112 (78.9)	<0.001
CABG, *n* (%)	186 (35.1)	13 (9.2)	<0.001

CABG, coronary artery bypass grafting; Some patients underwent multiple procedures; therefore, percentages may not sum to 100%.

### 3.4 Outcomes

#### 3.4.1 Mortality

##### 3.4.1.1 All-cause mortality during hospitalization

All-cause mortality during hospitalization differed significantly between SVS/STS-A and SVS/STS-B0 patients (Table 5). In the overall cohort (SVS/STS-A group, *n* = 743; SVS/STS-B0 group, *n* = 188), the SVS/STS-A group demonstrated significantly higher early and in-hospital mortality: 24 h mortality (9.8% vs. 1.1%, *P* < 0.001), 48 h mortality (12.9% vs. 2.1%, *P* < 0.001), and mortality during hospitalization (24.2% vs. 14.4%, *P* = 0.004). When stratified by treatment modality, outcomes diverged further. In the non-surgery subgroup (SVS/STS-A, *n* = 212; SVS/STS-B0, *n* = 35), the SVS/STS-A group demonstrated markedly elevated early and in-hospital mortality: 24 h mortality (31.6% vs. 5.7%, *P* = 0.002), 48 h mortality (38.7% vs. 8.6%, *P* < 0.001), and in-hospital mortality (49.5% vs. 8.6%, *P* < 0.001).In the open surgery subgroup (SVS/STS-A, *n* = 530; SVS/STS-B0, *n* = 142), rates of early and in-hospital all-cause mortality did not differ significantly: 24 h mortality (1.1% vs. 0%, *P* = 0.203), 48 h mortality (2.6% vs. 0.7%, *P* = 0.165), and in-hospital mortality (14.0% vs. 16.9%, *P* = 0.378).

**Table 5:** Mortality.

Variable	SVS/STS-A	SVS/STS-B0	*P-*value
Total cohort	*n* = 743	*n* = 188	
24 h mortality, *n* (%)	73 (9.8)	2 (1.1)	<0.001
48 h mortality, *n* (%)	96 (12.9)	4 (2.1)	<0.001
In-hospital mortality, *n* (%)	180 (24.2)	27 (14.4)	0.004
1-year all-cause mortality, (%)	25.9%	15.2%	0.0005
3-year all-cause mortality, (%)	31.7%	17.8%	0.0005
Open surgery group	*n* = 530	*n* = 142	
24 h mortality, *n* (%)	6 (1.1)	0 (0)	0.203
48 h mortality, *n* (%)	14 (2.6)	1 (0.7)	0.165
In-hospital mortality, *n* (%)	74 (14.0)	24 (16.9)	0.378
1-year all-cause mortality, (%)	14.9%	17.4%	0.540
3-year all-cause mortality, (%)	18.7%	20.7%	0.540
Non-surgery group	*n* = 212	*n* = 35	
24 h mortality, *n* (%)	67 (31.6)	2 (5.7)	0.002
48 h mortality, *n* (%)	82 (38.7)	3 (8.6)	<0.001
In-hospital mortality, *n* (%)	105 (49.5)	3 (8.6)	<0.001
1-year all-cause mortality, (%)	54.7%	8.6%	<0.001
3-year all-cause mortality, (%)	64.4%	8.6%	<0.001

##### 3.4.1.2 Mid-Term prognosis

**Figure 3:**
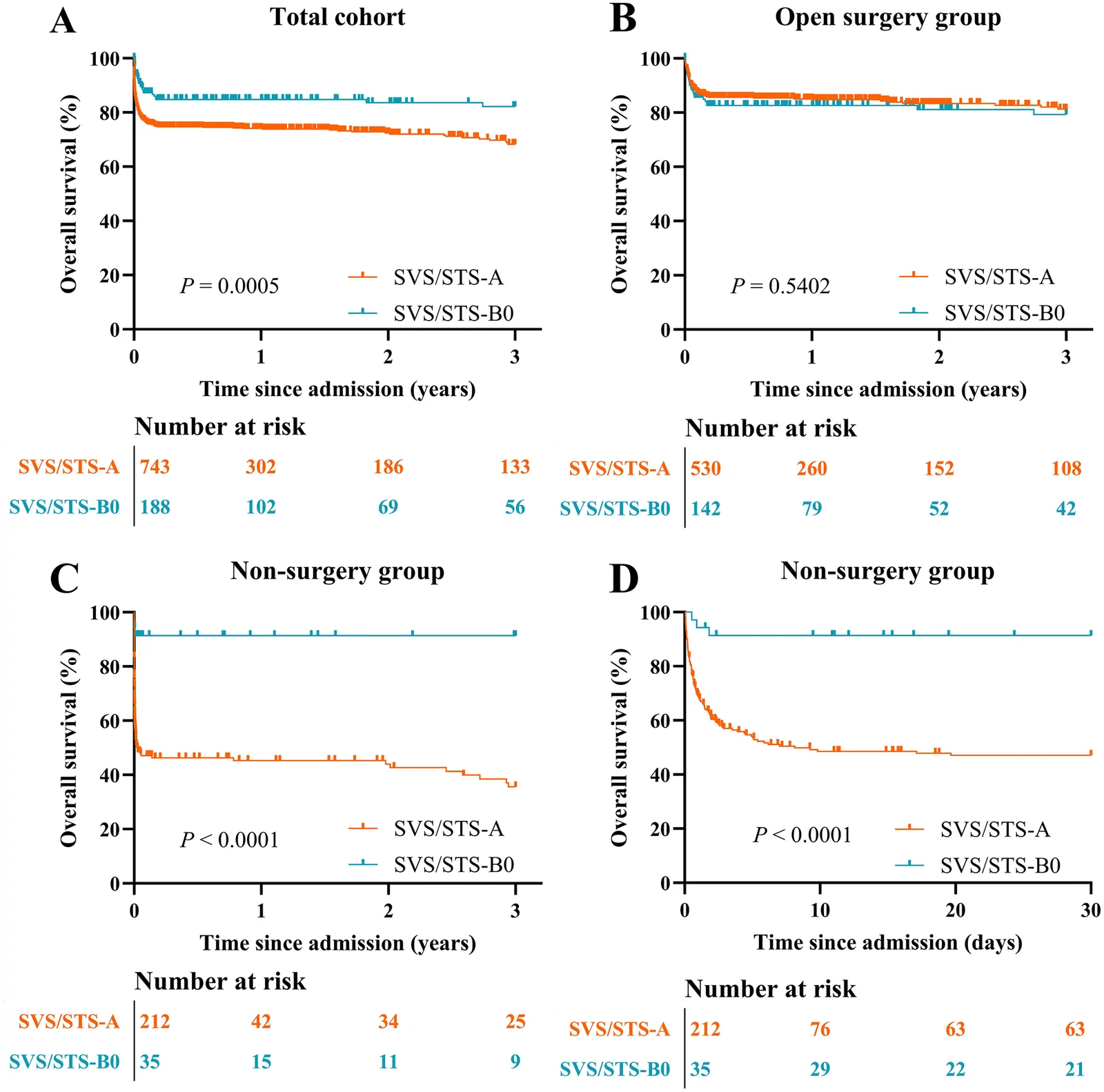
Kaplan–meier survival curves for all-cause mortality in the total cohort **(A)**, the open surgery subgroup **(B)**, and the non-surgery subgroup **(C,D)**. Log-rank test was used for comparison.SVS/STS, Society for Vascular Surgery and Society of Thoracic Surgeons.

Survival status was followed for up to 3 years post-admission via outpatient review or telephone, with Kaplan–Meier curves presented (Figure 3). In the overall cohort, median follow-up was 1.2 years (95% CI: 1.0–1.4). The SVS/STS-A group had higher 1-year (25.90% vs. 15.20%) and 3-year (31.70% vs. 17.80%) all-cause mortality, with a considerably higher risk of death (HR = 1.97, 95% CI: 1.44–2.69, *P* = 0.0005; Figure 3A). In the open surgery subgroup, median follow-up was 1.3 years (95% CI: 1.1–1.5). 1-year (14.9% vs. 17.4%) and 3-year (18.7% vs. 20.7%) mortality were similar between groups (HR = 0.87, 95% CI: 0.55–1.38, *P* = 0.540; Figure 3B). In the non-surgery subgroup, median follow-up was 0.7 years (95% CI: 0.28–1.1). The SVS/STS-A group experienced substantially higher 1-year (54.7% vs. 8.6%) and 3-year (64.4% vs. 8.6%) mortality, with a 9-fold increased death risk (HR = 9.01, 95% CI: 5.68–14.28, *P* < 0.001; Figure 3C).

Surgical intervention mitigated the early mortality hazard of the SVS/STS-A anatomy. The survival disparity was driven predominantly by excessive early mortality (within 30 days) in non-surgical SVS/STS-A patients, whereas SVS/STS-B0 patients maintained stable survival beyond 1 year (Figure 3D).

#### 3.4.2 Comparison of in-hospital complications and length of stay

In the overall cohort, the SVS/STS-A group had considerably higher rates of in-hospital aortic rupture (12.9% vs. 4.3%, *P* < 0.001) and cardiac arrest (15.9% vs. 5.9%, *P* < 0.001), but a reduced median length of hospitalization (18 vs. 24 days, *P* < 0.001). The incidence of massive pericardial hemorrhage, stroke, spinal cord ischemia, acute renal failure, and prolonged mechanical ventilation (>48 h) showed no substantial difference between groups (all *P* > 0.05) (Table 6).

**Table 6:** In-hospital complications and length of stay.

Variable	SVS/STS-A	SVS/STS-B0	*P*-value
Total cohort	*n* = 743	*n* = 188	
In-hospital Complications, *n* (%)			
Aortic rupture	96 (12.9)	8 (4.3)	<0.001
Cardiac arrest	118 (15.9)	11 (5.9)	<0.001
Massive pericardial hemorrhage	120 (16.2)	28 (14.9)	0.674
Stroke	147 (19.8)	39 (20.7)	0.769
Spinal cord ischemia	42 (5.7)	13 (6.9)	0.512
Acute renal failure	86 (11.6)	15 (8.0)	0.157
Prolonged MV (>48 h), %	45.2%	43.6%	0.773
Hospital Stay (days), median (IQR)	18 (1–113)	24 (1–85)	<0.001
Open surgery group	*n* = 530	*n* = 142	
In-hospital Complications, *n* (%)			
Aortic rupture	39 (7.4)	7 (4.9)	0.309
Cardiac arrest	30 (5.7)	9 (6.3)	0.759
Massive pericardial hemorrhage	94 (17.7)	27 (19.0)	0.752
Stroke	103 (19.4)	27 (19.0)	0.910
Spinal cord ischemia	32 (6.0)	11 (7.7)	0.46
Acute renal failure	81 (15.3)	14 (9.9)	0.099
Prolonged MV (>48 h), %	50.7%	45.6%	0.355
Hospital Stay (days), median (IQR)	20 (1–113)	24 (2–93)	0.002
No-surgery group	*n* = 212	*n* = 35	
In-hospital Complications, *n* (%)			
Aortic rupture	57 (26.9)	1 (2.9)	0.002
Cardiac arrest	88 (41.5)	2 (5.7)	<0.001
Massive pericardial hemorrhage	26 (12.3)	0 (0)	0.029
Stroke	44 (20.8)	9 (25.7)	0.508
Spinal cord ischemia	9 (4.2)	1 (2.9)	0.699
Acute renal failure	4(1.9)	1(2.9)	0.706

Between-group comparison of postoperative complication rates in the open surgery stratum showed no substantial difference (*P* > 0.05). The median length of stay remained shorter in the SVS/STS-A group (20 vs. 24 days, *P* = 0.002).

In the non-surgery subgroup, the SVS/STS-A group exhibited significantly higher rates of in-hospital aortic rupture (26.9% vs. 2.9%, *P* = 0.002), cardiac arrest (41.5% vs. 5.7%, *P* < 0.001), and massive pericardial hemorrhage (12.3% vs. 0%, *P* = 0.029). No substantial differences were observed in rates of stroke, spinal cord ischemia, or acute renal failure (all *P* > 0.05).

### 3.5 Risk factors for outcomes in the SVS/STS-A vs. SVS/STS-B0 cohorts

**Figure 4:**
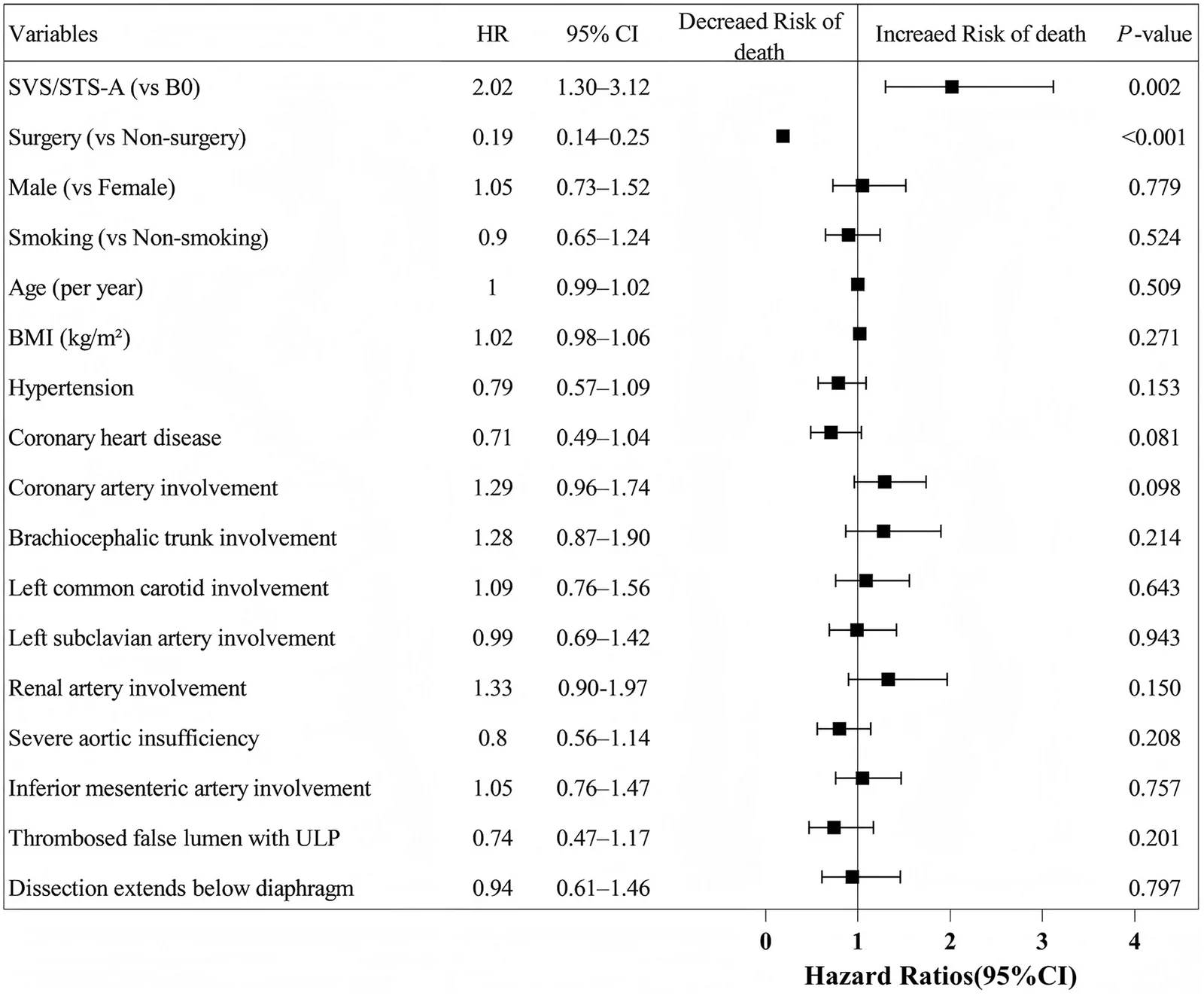
Multivariable Cox regression model of in-hospital mortality for SVS/STS-A vs. SVS/STS-B0.

We controlled for potential confounding factors such as sex, age, underlying diseases, aortic anatomical characteristics, and treatment modality in the multivariable Cox regression analysis (Figure 4). The results showed that SVS/STS-A classification (compared with SVS/STS-B0) was an independent risk factor for in-hospital mortality (HR = 1.69, 95% CI: 1.09–2.61, *P* = 0.019). Meanwhile, undergoing surgical treatment emerged as the strongest protective factor (HR = 0.18, 95% CI: 0.13–0.24, *P* < 0.001). Other factors, such as coronary artery involvement, showed a trend toward increased risk but did not reach statistical significance.

### 3.6 Outcomes of SVS/STS-I patients

Although this study primarily focused on the comparison between SVS/STS-A and SVS/STS-B0 dissections, we additionally analyzed the clinical outcomes of the SVS/STS-I cohort (*n* = 113; Table 7). Among these patients with unidentifiable primary entry tears, 53 underwent non-surgical management and 60 underwent surgical intervention. The non-surgical group exhibited significantly higher 48 h mortality (11.3% vs. 1.7%, *P* = 0.05) and in-hospital mortality (26.4% vs. 8.3%, *P* = 0.01) compared with the surgical group.

**Table 7:** SVS/STS-I mortality.

Variable	Non-surgery group (*n* = 53)	Surgery group (*n* = 60)	Total cohort (*n* = 113)	*P*-value
Mortality, *n* (%)				
24 h	2 (3.8)	0 (0)	2 (1.8)	0.218
48 h	6 (11.3)	1 (1.7)	7 (6.2)	0.05
In-hospital	14 (26.4)	5 (8.3)	19 (16.8)	0.01

*P* values are for comparison between surgery and non-surgery groups. The total cohort was not included in statistical comparisons.

### 3.7 Outcomes of patients with missing incomplete CTA data

Among the 2,297 patients excluded due to incomplete CTA data, 1,398 had Stanford type A and 899 had Stanford type B acute aortic dissection based on discharge diagnosis. Mortality outcomes stratified by Stanford type and treatment modality are shown in Table 8. In this population, Stanford type A patients had significantly higher early and in-hospital mortality compared to type B patients, consistent with the known natural history of the disease.

**Table 8:** Mortality outcomes in AAD patients without complete CTA data, stratified by Stanford type and treatment group.

Variable	Stanford-A	Stanford-B	*P-*value
Total cohort	*n* = 1,398	*n* = 899	
24 h mortality, *n* (%)	149 (10.7)	10 (1.1)	<0.001
48 h mortality, *n* (%)	209 (14.9)	17 (1.9)	<0.001
In-hospital mortality, *n* (%)	368 (26.3)	39 (4.3)	<0.001
Surgery group	*n* = 973	*n* = 537	
24 h mortality, *n* (%)	11 (1.1)	1 (0.2)	0.048
48 h mortality, *n* (%)	30 (3.1)	5 (0.9)	0.008
In-hospital mortality, *n* (%)	138 (14.2)	16 (3.0)	<0.001
Non-surgery group	*n* = 425	*n* = 362	
24 h mortality, *n* (%)	138 (32.5)	9 (2.5)	<0.001
48 h mortality, *n* (%)	179 (42.1)	12 (3.3)	<0.001
In-hospital mortality, *n* (%)	230 (54.1)	23 (6.4)	<0.001

AAD, acute aortic dissection.

To evaluate potential selection bias, we compared mortality between the study cohort (ATAAD with complete CTA data, *n* = 1,044) and the excluded ATAAD patients (*n* = 1,398) (Table 9). Excluded patients had higher overall 24 h (10.7% vs. 7.4%, *P* = 0.006), 48 h (14.9% vs. 10.2%, *P* < 0.001), and in-hospital mortality (26.3% vs. 21.6%, *P* < 0.001). Notably, among patients undergoing surgery, mortality rates were similar (14.2% vs. 14.0%, *P* = 0.904), whereas non-surgically managed excluded patients had markedly higher mortality (54.1% vs. 40.7%, *P* < 0.001). These findings suggest that patients with missing incomplete CTA data, particularly those managed non-operatively, may represent a sicker cohort, potentially introducing selection bias toward better outcomes in the main study population.

**Table 9:** Comparison of mortality between ATAAD patients with complete CTA data and those with missing CTA data.

Variable	Missing CTA group	Complete CTA group	*P-*value
Total cohort	*n* = 1,398	*n* = 1,044	
24 h mortality, *n* (%)	149 (10.7)	77 (7.4)	0.006
48 h mortality, *n* (%)	209 (14.9)	107 (10.2)	<0.001
In-hospital mortality, *n* (%)	368 (26.3)	226 (21.6)	<0.001
Surgery group	*n* = 973	*n* = 744	
24 h mortality, *n* (%)	11 (1.1)	6 (0.8)	0.502
48 h mortality, *n* (%)	30 (3.1)	16 (2.2)	0.236
In-hospital mortality, *n* (%)	138 (14.2)	104 (14.0)	0.904
Non-surgery group	*n* = 425	*n* = 300	
24 h mortality, *n* (%)	138 (32.5)	71 (23.7)	0.010
48 h mortality, *n* (%)	179 (42.1)	91 (30.3)	0.001
In-hospital mortality, *n* (%)	230 (54.1)	122 (40.7)	<0.001

Complete CTA group refers to Stanford type A patients with full aortic CTA data (the main study cohort); Missing CTA group refers to Stanford type A patients excluded due to lack of complete CTA data.

## 4 Discussion

This study applied the SVS/STS classification (8) to a single-center cohort of ATAAD patients, comparing the SVS/STS-A and SVS/STS-B0 groups. It confirms significant intergroup differences in baseline characteristics, anatomical features, treatment strategies, and natural history. Our analysis revealed two pivotal findings: first, SVS/STS-A dissection carries a catastrophic early mortality risk (3), starkly highlighted when pre-intervention deaths are accounted for; second, this classification effectively guides divergent surgical strategies—root-oriented for SVS/STS-A vs. arch/distal-focused for SVS/STS-B0 (10). By distinguishing between antegrade (SVS/STS-A) and retrograde (SVS/STS-B0) primary tears, this classification provides a novel and practical framework for tailoring the management of ATAAD.

Baseline characteristics differed between patients with SVS/STS-A and SVS/STS-B0 dissections. The SVS/STS-B0 patients exhibited a more typical atherosclerotic risk factor profile (11), characterized by a higher male predominance, higher smoking rates, and a greater prevalence of hypertension. The primary entry tear in SVS/STS-B0 dissections was most frequently located in Zone 3 (42.1%), an area distal to the left subclavian artery origin where vascular wall shear stress is higher (12, 13). Chronic hypertension in this region may predispose to intimal tearing. In contrast, entry tears in SVS/STS-A dissections were exclusively located in the ascending aorta. The pathogenesis of this subtype is likely more closely associated with medial degenerative changes, genetic factors, or distinct inflammatory mechanisms (14). These observed differences suggest distinct underlying pathophysiological pathways.

This study, along with the research by Ikeno et al. (15), found significant differences in the anatomical features of aortic dissection exist between the SVS/STS-A and SVS/STS-B0 groups, which directly drive individualized surgical strategies. In SVS/STS-A dissections, where the primary entry tear is located in the ascending aorta, catastrophic proximal structural damage occurs: there is a significantly higher rate of coronary ostia involvement (this study: 42.8% vs. 25.5%, *P* < 0.001; Ikeno et al.: 7.1% vs. 3.7%) and a higher incidence of severe aortic regurgitation (this study: 31.6% vs. 10.6%, *P* < 0.001; Ikeno et al.: 30.2% vs. 11.7%). Consequently, the rates of aortic root replacement (this study: 36.8% vs. 7.7%, *P* < 0.001; Ikeno et al.: 11.9% vs. 5.6%), aortic valve replacement (35.7% vs. 9.9%, *P* < 0.001), and coronary artery bypass grafting (this study: 35.1% vs. 9.2%, *P* < 0.001; Ikeno et al.: 9.3% vs. 6.8%) were all significantly higher in SVS/STS-A patients compared to SVS/STS-B0 patients. In contrast, SVS/STS-B0 dissections, which involve retrograde extension to the ascending aorta, have relatively preserved aortic root anatomy but exhibit extensive distal dissection propagation (rate of extension below the diaphragm: 87.2% vs. 69.6%, *P* < 0.001). Therefore, the surgical focus for SVS/STS-B0 patients shifts toward more extensive aortic arch replacement (78.2% vs. 65.1%, *P* = 0.012) and the use of stented elephant trunk procedures (78.9% vs. 59.1%, *P* < 0.001). This strategy aims to promote distal true lumen remodeling, address the primary entry tear, and prevent the development of late aneurysmal degeneration. This observation aligns with findings from a Chinese multicenter registry study, which reported high application rates of total arch replacement (88.7%) and the stented elephant trunk technique (75.6%) in patients with acute type A aortic dissection (16). This trend may be associated with the widespread adoption and former standard status of the Sun's procedure in China.

In this study, 7.2% (11/153) of SVS/STS-B0 patients who received surgical intervention underwent endovascular therapy alone. Notably, there were no in-hospital deaths among these 11 patients, providing preliminary evidence for the SVS/STS classification guiding minimally invasive treatment strategies. This outcome is consistent with the findings reported by Higashigawa et al., who demonstrated a low mortality rate (30-day mortality of only 3%) with TEVAR for retrograde type A aortic dissection (17). While our data suggests feasibility in highly selected cases, the small sample size precludes definitive conclusions. Future prospective studies are needed to establish precise anatomical criteria for endovascular management in SVS/STS-B0 dissections. However, it must be emphasized that the cases in our cohort underwent rigorous selection. All 11 patients met the following criteria: (1) the entry tear was located in zone 3–5 (distal to the left subclavian artery); (2) the extent of ascending aortic involvement was limited; and (3) there was no sinus of Valsalva involvement, the coronary arteries were intact, and valve function was normal. Lopez-Marco et al. cautioned that retrograde dissections are often associated with a higher risk of lower limb ischemia (27%), which TEVAR may not resolve (18). Therefore, while the SVS/STS classification offers the possibility of minimally invasive treatment, decision-making must be cautious and based on detailed anatomical assessment. Currently, open surgery remains the mainstream approach.

The key finding of this study is the revelation of a catastrophic pre-intervention mortality risk in SVS/STS-A patients, which has been severely underestimated in previous studies based primarily on surgical cohorts. Previous studies, Ikeno et al. only enrolled patients who underwent open surgery, reporting no significant difference between the SVS/STS-A and B0 groups in operative mortality (13.9% vs. 14.8%, *P* = 0.778) or long-term survival (10-year survival: 59.3% vs. 54.5%, *P* = 0.277), although the B0 group had a significantly higher rate of distal reintervention (10-year freedom from reintervention: 76.2% vs. 65.3%, *P* = 0.008) (15). However, by excluding pre-intervention deaths, such studies introduce significant survivor bias and a consequent underestimation of the true lethality of SVS/STS-A dissection. In contrast, by including all patients in our cohort, our study found that the SVS/STS-A group had significantly higher 24 h mortality (9.8% vs. 1.1%, *P* < 0.001), in-hospital mortality (24.2% vs. 14.4%, *P* = 0.004), and 3-year all-cause mortality (31.7% vs. 17.8%, *P* = 0.0005) in the overall cohort, providing a more accurate reflection of the lethal nature of SVS/STS-A dissections. This difference was particularly pronounced in the non-operative management subgroup, where SVS/STS-A patients had a 24 h mortality rate as high as 31.6% and an in-hospital mortality rate of 49.5%, compared to only 5.7% and 8.6%, respectively, for the SVS/STS-B0 group.When the primary entry tear is located in the ascending aorta, the dissection flap can instantaneously obstruct coronary arteries, cause severe aortic regurgitation, or lead to cardiac tamponade, precipitating hemodynamic collapse. Notably, in the surgical subgroup, the in-hospital mortality in our study (14.0% vs. 16.9%) was similar to that reported by Ikeno et al., further confirming that previous studies underestimated the early risk of the SVS/STS-A group by excluding pre-intervention deaths. This finding has critical triage implications: patients with SVS/STS-A dissection warrant immediate activation of the highest level of emergency response and urgent transfer to the operating room, whereas patients with SVS/STS-B0, while still requiring urgent surgery, have a relatively wider decision-making window.

The SVS/STS classification thus moves beyond mere anatomical description towards a clinically actionable framework, facilitating risk communication, emergency triage, and personalized surgical planning.

## 5 Limitations

This study has several inherent limitations. First, it is a single-center retrospective analysis, which may be subject to selection bias. Second, treatment strategies could be influenced by non-medical factors such as economic considerations and family preferences, which are difficult to quantify. Third, the identification of the primary entry tear location relied on manual interpretation of CTA images, introducing a potential for error. Fourth, some patients were excluded due to incomplete data, which may introduce selection bias; therefore, our findings should be interpreted with caution.Finally, the median follow-up period of 1.2 years is insufficient to adequately evaluate long-term outcomes, including aortic remodeling and reintervention rates.

## 6 Conclusion

The SVS/STS classification is an effective tool for guiding precision in the diagnosis and treatment of ATAAD. The SVS/STS-A dissection carries a catastrophic early mortality risk, underscoring the imperative for emergent surgical intervention. In contrast, while the SVS/STS-B0 dissections are frequently associated with extensive distal involvement, they offer a relative flexibility in the surgical time window, and selected cases may be candidates for endovascular therapy. This classification system demonstrates substantial clinical utility for emergency triage, surgical decision-making, and prognostic assessment.

## Data Availability

The raw data supporting the conclusions of this article will be made available by the authors, without undue reservation.
